# A case report on a child with fracture and dislocation of the upper cervical spine accompanied by spinal cord injury

**DOI:** 10.1097/MD.0000000000029717

**Published:** 2022-07-29

**Authors:** Jiayu Zeng, Hua Jiang, Yingquan Zhuo, Yongkang Xu, Zhigang Deng

**Affiliations:** a School of Clinical Medicine, Guizhou Medical University, Guiyang City, Guizhou Province, People’s Republic of China; b Department of Pediatric Surgery, Affiliated Hospital of Guizhou Medical University, Guiyang City, Guizhou Province, People’s Republic of China; c The Second Affiliated Hospital of Nanchang University, Nanchang University, Nanchang City, Jiangxi Province, People’s Republic of China.

**Keywords:** dislocation, fracture, locked facets, spine cord injuries, upper cervical spine

## Abstract

**Rationale:**

This study describes an 8-year-old boy with a C2 fracture and dislocation with a left C2–C3 articular process interlocking and spinal cord injury who underwent open reduction and internal fixation using the posterior cervical approach and achieved satisfactory results.

**Patient concerns:**

An 8-year-old boy underwent an emergency transfer from a previous hospital after a car accident.

**Diagnoses:**

Axial fracture and dislocation with spinal cord injury (American Spinal Injury Association grade C), traumatic shock, brain contusion, intracranial hemorrhage, mandibular fracture, pulmonary contusion and hemorrhage, left vertebral artery stenosis, and multiple fractures throughout the body. Radiological examination revealed a fracture of the lower edge of the C2 vertebral body, fourth-degree anterior spondylolisthesis of the C2 vertebral body, interlocking of the left C2–C3 articular processes, widening of the C2–C3 vertebral space, and occlusion of the V1 and 2 segments of the left vertebral artery.

**Interventions:**

The boy was immediately intubated and transferred to the pediatric intensive care unit for rescue treatment. However, the reduction was unsuccessful with 2 weeks of cranial traction. Thus, an open reduction was performed under general anesthesia. One month after the surgery, the boy was discharged from the hospital on foot after rehabilitation treatment.

**Outcomes:**

The boy was discharged from the hospital 1 month after surgery. At the 8-month follow-up, a radiological examination showed that the corrected C2 vertebral body fracture and dislocation were satisfactorily reduced, and the spinal cord was adequately decompressed. The internal fixation position was also good, and the spinal sequence had recovered well. In summary, except for the muscle strength of the right upper limb, which was slightly worse, the other clinical symptoms were significantly improved.

**Lessons:**

In treating cervical fracture and dislocation with unilateral facet lock, the posterior open reduction of pedicle screw and lateral mass screw internal fixation achieved satisfactory results. Consequently, treating complex cervical spine injuries in children requires an accurate diagnosis and careful treatment strategy.

## 1. Introduction

Cervical spine injuries (CSIs) in children are relatively rare, accounting for only 1% to 2% of the total number of injuries.^[1–4]^ However, CSI accounts for the majority, 60% to 80%,^[5]^ of children’s spinal injuries. Simple injuries to the upper cervical spine account for 37% of CSIs, with a mean age of 9.42 ± 5.12 years.^[6]^ CSI in young children is usually caused by motor vehicle accidents, falls, or child abuse.^[7–11]^ Although CSI is rare in young children, it can lead to severe complications once it occurs. CSI in children accounts for 1% to 19% of all cases of spinal cord injury (SCI), which are most common in the neck.^[12–18]^ Children with a better SCI prognosis have more substantial repair potential than adults.^[5, 18–22]^ Because there is no unified standard for the diagnosis and treatment of CSI in children,^[23,24]^ treatment is extremely challenging. This paper reports a case of C2 fracture and dislocation with left C2–C3 facet locking accompanied by SCI treated in the Affiliated Hospital of Guizhou Medical University in 2020.

## 2. Case report

**Figure 1.**
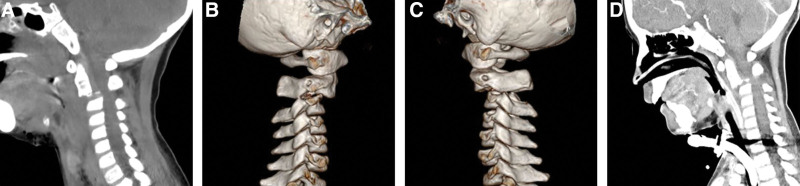
Three-dimensional CT (A, B and C) shows the fractures of the lower margin and IV° anterior spondylization of the C2 vertebral body, the fractures of the upper margin of the C3 vertebra and right transverse process, and left C2–C3 facet locking, while (D) shows no significant reduction in the C2 fracture-dislocation after 2 weeks of traction.

**Figure 2.**
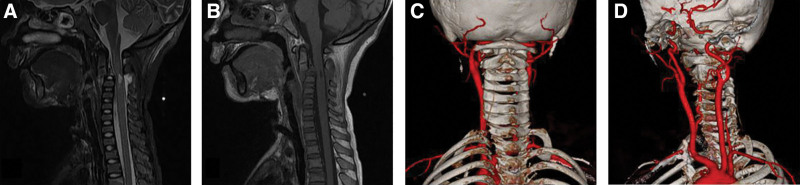
Preoperative cervical MRI (A and B) showed C2–C3 level spinal cord injury, C2 lower margin fracture, C2 IV° anterior slippage, and C2–C3 space widening. CTA enhancement of the cervical vessels (C and D) showed that the left vertebral artery V1 and 2 segments were occluded. The communication branch between the left external carotid artery and the left vertebral artery was developed in the C2 plane, and there was no vertebral artery riding.

The child, male, 8 years old, was injured in a car accident and admitted to a local hospital. He was intubated immediately because he could not breathe spontaneously. Because of the critical condition, the child was transferred to the pediatric intensive care unit of our hospital for rescue treatment on the same day. The diagnosis was as follows: C2 fracture, dislocation, facet locking, and SCI (Fig. 1A–C); traumatic shock; brain contusion and laceration; intracranial hemorrhage; left vertebral artery stenosis (Fig. 2C, D); mandibular fracture; pulmonary contusion and pulmonary hemorrhage; and left rib fracture. The results of the physical examination showed vague, simple responses, no spontaneous breathing during endotracheal intubation, lack of coordination, no coordination during neurologically sensory plane physical examination; right upper limb: muscle strength grade 0, muscle tension grade 0; left upper limb: muscle strength grade 2, muscle tension grade 2; lower extremity activity was not bad, with muscle strength grade 3, hypertonic muscle, no muscular atrophy, and hypertrophy; no superficial sensation; corneal reflex and light reflex existed; abdominal wall reflex, testicular reflex, and anal reflex not elicited; knee–tendon reflex was hyperactive; neck stiffness not checked; Hoffmann sign not elicited, Babinski sign (+), Oppenheim sign (+), Chaddock sign (+), and Gordon sign (+). The muscle strength results after specialist examination are shown in Table 1 below.

**Table 1:** The muscle strength results after specialist examination.

**Muscle strength**	**On admission**	**Preoperative**	**On discharge**	**11 mo postoperative**
Biceps brachii	L3, R2	L3, R2	L5, R3–	L5, R4
Extensor carpi radialis longus	L3, R0	L3, R0	L5, R3–	L5, R4
Finger extensor flexor	L3, R1	L3, R1	L5, R3–	L5, R4
Abductor little finger	L3, R1	L3, R1	L5, R3–	L5, R4
Triceps brachii	L3, R1	L3, R1	L5, R3–	L5, R4
Iliac psoas	L3, R3	L3, R3	L3+, R3+	L5, R5
Quadriceps	L3+, R3+	L3+, R3+	L3+, R3+	L5, R5
Tibialis anterior muscle	L3, R3	L3, R3	L3+, R3+	L5, R5
Long extensor muscle	L2, R2	L2, R2	L3+, R3+	L5, R5
Calf triceps	L2, R2	L2, R2	L3+, R3+	L5, R5

Skull traction was performed on admission, but the reduction was unsuccessful (Fig. 1D). After a comprehensive evaluation and active treatment, the child’s condition was stabilized. Fifteen days after the injury, the child was transferred to our department. After eliminating surgical contraindications, posterior cervical open reduction + C2/3/4 pedicle screw and lateral mass screw internal fixation were performed. The surgical procedure was as follows:

A push-in position was taken, cranial traction with a traction weight of 2 kg, and the cervical vertebra was maintained during surgery. The C2–C6 to the neutral cutting length of 10 cm was taken, the skin was cut subcutaneously, the semispinalis capitis was cut open, the C2–C4 double laminate was revealed, spinous processes, and the C2–C3 joints was blocked in left side. The reset of the skull traction was presented, but because of the difficulties in levering to reset, partial resection was taken to reset. About 2 mm from the C2 on the top joint knob, the middle of the side block was pulled upside down, the vector plane was 40°, and the side angle of the angle was opened to the head-side angle; the probe detected the intact of the vertebrae, no damage. The 2 sides were screwed into two 3.5 mm wide, length 2.0 cm universal pedicle nails. C3 on the right side of the upper quadrant point was open, and the sagittal plane was 45°; the angle of the head side was opened to the head side; the probe explored the rupture of the inner wall of the right vertebrae root. C3 pedicle nails are difficult to fix, we selected the side block screw-fixing in the inner side in the right side block, topping up, tilting 45° openings, the probe ensured the integrity of the front wall, the rear wall, the inner wall and the outer side wall, screwed into a 3.5-mm wide, a length of 2.5-cm universal pedicle nails, and the left side block is broken, and the side block screw fixation is not considered. A fixed C4 vertebral body, in the inner side in the bilateral side block, tilted up 30°, tilted 40° openings, the probe ensured the integrity of the front wall, the rear wall, the inner wall, and the outer side wall, then the 2 suitable size universal vertebrae nails were screwed into to fixation. The pedestrian and articulating bone cortex in C2–C4 were grinded, then we planted about 2.0 g of pine bone was taken in the left anterior superior iliac spine and 6.0 g of the same kind of allogeneic bone. The blood supply was 100 mL.

The prone position was taken; skull traction was performed with a traction weight of 2 kg; and the cervical spine was kept in a neutral position during the operation.

**Figure 3.**
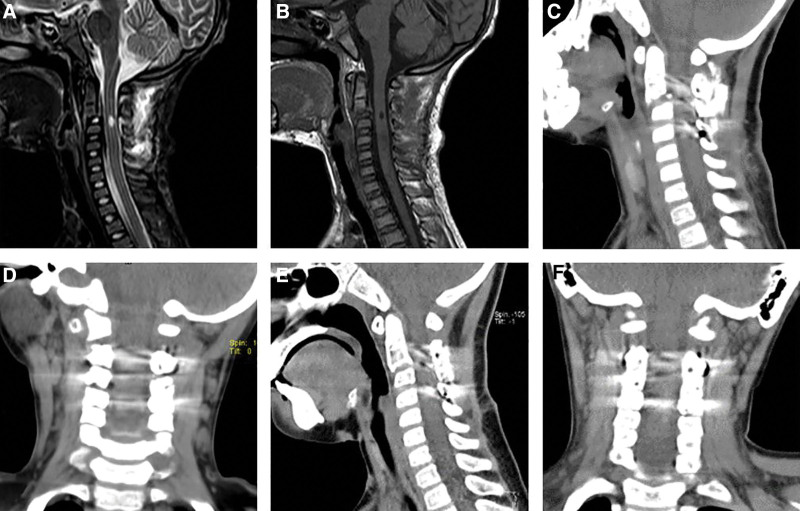
The postoperative cervical MRI (A and B) showed that the spinal cord injury at the C2/3 intervertebral space level was better than before. The postoperative cervical CT (C and D) showed that C2 vertebral body forward slippage was significantly improved, spinal canal stenosis in the corresponding segment was reduced, the cervical spine sequence was well recovered, and screws were accurately implanted into the pedicle.

The child was able to walk 2 weeks after surgery and regained muscle strength 1 month after surgery. During the 8-month follow-up, the lower limb function and urination/defecation function were completely regained, the right upper limb function was significantly improved (Table 1), and no other complications were found. The computed tomography showed the spine was in good shape with intact screw positions (Fig. 3E, F).

## 3. Discussion

Pediatric spinal traumas (PSTs) are not common, and the type of trauma and spinal segment affected varies with age.^[5,6]^ CSI mainly occurs in young children, and the most commonly affected spine in PST is the cervical spine (60%–80%).^[5,25,26]^ Upper cervical injury usually refers to damage to C1–C2, which mainly involves fractures; torn ligament and dislocation caused by violence on the atlanto-axial vertebral and its subsidiary structure, including the dislocation between atlas and the occipital; transverse ligament damage; atlanto-axial joint dislocation; atlas fractures; posterior atlanto-axial dentate condyle fractures; posterior atlanto-axial traumatic slippage; lateral fracture; vertebral body fracture; and anterior dislocation of the fracture and multiple injuries. Simple upper cervical injuries account for 37% of all cervical injuries, and the mean age of upper cervical injuries is 9.42 ± 5.12 years. The incidence of upper CSI decreases with the increase in patients’ age; the incidence of simple upper CSI was 72.7% in children <3 years old, 47.8% in children between 3 and 8 years old, and 29% in children >8 years old.^[6]^

The cervical spine of children of different ages presents different anatomical characteristics from that of adults. The anatomical structure of the cervical spine of children is relatively immature. Before the age of 8, the cervical spine is characterized by a flat occipital condyle, wedge-shaped vertebra, horizontal facet joint, and relatively large and underdeveloped muscles of the head and neck.^[8,10,27,28]^ Ossification begins around 8 years old and is completed at 12 to 13 years old.^[29]^ During this maturation, the fulcrum of the cervical motion migrates downward to the C5–C6 level of the lower cervical spine.^[28]^ The anatomical and biomechanical characteristics of the immature cervical spine make the upper C1–C3 segments, especially the C2 segment, more vulnerable to injuries in young children, while adolescents tend to have more injuries in the lower cervical spine.^[8,10,29–31]^

Injury of the upper cervical spine is usually high-energy trauma,^[8,10,32,33]^ which is often accompanied by severe spinal cord and medulla oblongata injuries, resulting in respiratory arrest. Children with relatively large heads are prone to cervical flexion and extension injuries, leading to severe spinal injuries.^[34,35]^ In addition, because of the laxity of spinal ligaments and neck muscles, a child’s spine is highly flexible, extending up to 5 cm, whereas the spinal cord can only extend to 5 mm. The spinal cord is relatively fixed in comparison to the range of motion of the spine. Therefore, a spinal cord with more flexibility is more likely to result in SCI.^[36,37]^ The spinal cord, located in the upper cervical canal, is an important part of the central nervous system that is vulnerable to violence, while the integrity of the upper cervical bony structure is essential for survival and function.

Motor vehicle accident is the most common cause of CSI in children. Although the incidence of spinal trauma (PST) in children is lower than in adults, children are more likely to have neurological impairment, and the death rate related to cervical spine trauma is significantly higher among children. The risk of multisystem damage in children is also high.^[38,39]^ The most common injury type in cervical vertebra injury is dislocation. Because fractures and dislocations do not cause height loss but can cause neurological defects, the classification of a nerve injury has nothing to do with height loss in the vertebral body.^[40]^

In this case, 15 days of cranial traction resulted in almost no reduction in cervical dislocation. Because of the lock of the left C2–C3 articular process, closed reduction was ineffective. So, open reduction and fixation were required to complete the computed tomography angiography examination of the carotid artery to exclude vertebral artery riding. However, the choice of internal fixation method was another difficult problem because cervical anterior titanium plate screws are not conducive to spinal growth. In addition, the anterior approach may increase the risk of infection and other complications after tracheotomy in children. Therefore, it is not suitable for the anterior approach. Cervical pedicle screw implantation in children is feasible because it can reduce the degree of fixation and fusion, has superior biomechanical properties, improved fusion rates, and external fixation is not needed. Both lateral mass fixation and pedicle screw fixation are technically challenging and carry the risk of serious complications if not performed properly.^[41]^

At the follow-up 11 months after the operation, the lower limb and urination/defecation functions were completely recovered, while right upper limb function had significantly improved (Table 1). The screws were implanted accurately as planned and the cervical sequence was well recovered. Eight months after the operation, 3-dimensional computed tomography image reconstruction showed a fusion of the C2–C4 vertebral (Fig. 3) and no developmental delay of the vertebral body and spinal canal. The effect of the posterior pedicle screws on the development of the upper cervical spine needs further long-term follow-up.

## Author contributions

Jiayu Zeng carried out the studies, participated in collecting data, and drafted the manuscript. Jiayu Zeng and Hua Jiang performed the statistical analysis and participated in its design. Yingquan Zhuo, Yongkang Xu, and Zhigang Deng helped to draft the manuscript. All authors read and approved the final manuscript.

Conceptualization: Jiayu Zeng, Yongkang Xu, Hua Jiang.

Data curation: Jiayu Zeng, Yingquan Zhuo, Zhigang Deng.

Formal analysis: Jiayu Zeng, Hua Jiang.

Investigation: Jiayu Zeng, Yongkang Xu.

Methodology: Hua Jiang.

Writing—original draft: Jiayu Zeng.

Writing—review and editing: Hua Jiang.
